# ‘Functional hyperthermia’: a historical overview

**DOI:** 10.1186/s13030-023-00292-3

**Published:** 2023-11-13

**Authors:** Mathieu Ginier-Gillet

**Affiliations:** Alumnus of the Grenoble Alpes Faculty of Medicine, La Tronche, France

**Keywords:** History of medicine, Fever of unknown origin, Functional somatic syndrome, Low-grade fever, Medically unexplained symptoms, Miscellaneous disorders

## Abstract

The management of low-grade fever in adults has not been codified. This gap is related not only to the numerous possible aetiologies but also to the difficulty of escaping the monocausal model of diseases. This article explores the complex issue of positive signs in ‘psychogenic fever’ through Reimann’s 1930s series. The discussion emphasises Canguilhem’s positions regarding vital signs and proposes (1) a semantic clarification of ‘habitual hyperthermia’ and (2) an amendment of the Belgian diagnostic criteria based on the concept of functional disorder. This paper also suggests following Peirce’s pragmatism in the face of an uncommon clinical picture.

*Between physiology and pathology there is no threshold.*

**Leriche**, The Normal and the Pathological by Canguilhem

*Only facts can express a sense, a class of names cannot.*

**Wittgenstein**, Tractatus Logico-Philosophicus

## Background

The modern concept of ‘functional hyperthermia’ (FH) is analogous to ‘habitual hyperthermia’ (HH), which is widespread in 20th-century medical literature [1].^1^ The term HH repeatedly appears in case series of fevers of unknown origin and is present in early American diagnostic algorithms [2, 3]. However, some have noted that the syndrome lacks a definition, and others have recommended abandoning the term [4, 5]. Nevertheless, even if HH appears outdated considering new diagnostic capabilities, the term can serve as a paradigm for addressing the complexity of persistent fever in general practice [6]. Indeed, most of the symptoms encountered in primary care are vague, and the level of somatisation in patients can be high [7]. The purpose of this paper is to examine the clinical aspects of FH/HH from a historical perspective.

## Sources of information

References were collected through the MEDLINE, Internet Archive, and Gallica databases. Special attention was given to work from Belgium and Japan, two countries leading research on fevers of unknown origin and psychogenic hyperthermia. Reimann’s^2^ 1930s articles were used to understand the concept of HH, especially because his publications led Petersdorf and Beeson^3^ to exclude HH from their cohorts [8, 9]. The most recent literature on FH was reviewed to identify similarities with Reimann’s cases. Furthermore, the commentary focuses^4^ on the views of Canguilhem (1904–1995), a French philosopher and physician, regarding ‘vital norms’ and their implications in daily practice [10, 11].

## Reimann’s position between the two world wars

The term HH first appeared in German literature in 1918. Moro observed that the temperature of some children was exaggerated in the second half of the day and reached 100.4°F (38.0 °C) without apparent cause [12]. He suggested a morbid predisposition^5^ and thus separated HH from ‘exercise hyperthermia’ (*bewegungshyperthermie*). His observations on temperature lability in children are consistent with those of Neff [13]. However, in 1924, Finkelstein hypothesised a postinfectious state, while Brünecke suggested that HH should be classified as a neurosis [14, 15].^6^ HH is therefore an ambiguous concept. The expression poorly differentiates between physiological 24-h temperature fluctuations of up to 2.4°F (1.3 °C) per day and more complex clinical situations in which possible infections and psychological disorders are involved [16]. This difficulty explains the issues Reimann faced: should the expression be reserved for healthy individuals with a higher average temperature (i.e. above the 95th percentile of a representative sample)? Or should HH be regarded as an entity?

From 1932 to 1936, Reimann analysed a series of sixteen cases of low-grade fever. Table 1 summarises the clinical features of these patients and focuses on Holló and Holló-Weil’s now outdated pharmacological method to identify subclinical tuberculosis [17–20]. Ultimately, the main clinical difference between the two groups proposed by Reimann is the higher symptom burden in cases of neurosis. Patients also have more active coping in the HH group and are somehow aware of the benign nature of their temperature. There are no negative consequences for their social relationships.

**Table 1:** Clinical features of patients with ‘habitual hyperthermia’ or ‘neurosis’ based on Reimann’s work

Case	Year	Name	Age, y	Duration of complaints, y	Bed rest, m	Fastigium, F°	Basic work-up^a^	Holló and Holló-Weil test^b^	Psychiatric evaluation	Actual perspective
Habitual Hyperthermia										
1	1933^c^	Miss B. E	26	21	NA	100.8	Yes	Positive	No	Long-term complication of measles
2	1936	Mrs M. Z	NA	4	NA	99.6	Yes	NA	No	Abdominal surgical complications
3	1936	Mrs M. A	28	4	NA	99.6	Yes	NA	No	Gynaecologic infection
4	1936	Miss J. V	17	2	NA	100	Yes	Positive	No	Chronic sinusitis
5	1936	Miss P. J	26	NA	NA	100.6	Yes	NA	No	Renal abscess
Neurosis^d^										
6	1932	Miss R. L	38	2	15	100.4	Yes	Positive	No	Munchausen's syndrome
7	1933	Miss K. U	35	6	5	99.5	Yes	Positive	No	Cerebellitis^e^
8	1934	Miss E. R	47	14	NA	100	Yes	Positive	No	Parasitic disease
9	1933	Miss R. R	22	2	NA	103.5	Yes	Negative	No	ARF recurrence
10	1935	Miss K. M	30	11	8	100	Yes	Positive	Yes	Virilising tumour
11	1935	Miss E. E	23	5	NA	100	NA	NA	No	PMDD
12	1935	Miss E. McG	15	5	NA	100.4	NA	NA	Yes^f^	ENT surgical complications

*Abbreviations*: *ARF* acute rheumatic fever, *ENT* ear, nose, throat, *NA* not available, *PMDD* premenstrual dysphoric disorder

*Notes*: ^a^ Basic workup ‘*included registration of several temperature readings daily for at least a month at repeated intervals, morphologic and numerical studies of blood cells, determination of the blood sedimentation rate, Wassermann reaction and various agglutinins, intradermal tests for tuberculosis and brucellosis, basal metabolic rate, constancy of body weight, and any special procedures as dictated by the individual case.*' ([...] p. 1090). ^b^ According to Holló and Holló-Weil, in HH, the temperature is depressed by certain narcotics (powdered opium) but not influenced by antipyretics (aminopyrine, a drug withdrawn from the U.S. market in 1970 due to the risk of agranulocytosis) [20]. ^c^ Date of the second admission to the university hospital. ^d^ Reimann uses the term neurosis to refer to personality disorders, but he does not quote any mainstream psychiatrist. ^e^ The downbeat nystagmus suggests a central nervous system lesion. ^f^ The psychiatrist mentioned a ‘*functional neurosis*.’ ([19] p. 1092)

In addition to the methodological problems (details of four cases are missing, only one case was followed up over five years, and the choice of thermometer is not specified), the articles state: (1) that Reimann dismissed HH from the spectrum of mental disorders; (2) that he never excluded the possibility of an infection or a rare event; and (3) that he questioned the validity of the HH concept because of the risk of medicalization of physiological reactions, as noted in the quote below:

The question may be raised whether the term “habitual hyperthermia” or any term need be applied to the type of patient described. There appears to be no more reason to do so than to apply the term “habitual bradycardia” to normal persons with an average pulse rate of 60 ([19] p. 1093).

With regard to more recent literature, this last point suggests (4) that Reimann was opposed to rest cure, despite Mitchell’s influence in Philadelphia; (5) that one of the reasons for hospitalisation may have been family pressure regarding marriage; (6) that temperature charts may have been influenced by hospital acclimation; and (7) given no ‘normothermia’ definition, certain measurements had led to cascade effects [21–25].

However, the key element of the series is the difficulty of establishing a boundary between HH and the concept of neurosis [26, 27]. In most of Reimann's cases, there is no reason to exclude a psychopathological process. The return to a ‘normal’ life of Miss B. E. (the main case, detailed in the three publications) and the discontinuity of her symptoms evokes what psychologist Janet called a ‘*banal neurosis*,’ in which ‘*certain higher operations, certain acts, certain perceptions are already suppressed or altered*.’ ([28] p. 393). Of course, Janet’s observations need to be tempered by Gilman’s^7^ account that the confinement of patients to bed must have caused iatrogenic symptoms [29, 30]. However, Kubie’s hypothesis best addresses the ‘*distinction between psychological illness and psychological health*.’ ([31] p. 176). According to Kubie, normality refers to plasticity, while neurosis refers to automaticity or to the ‘*freezing of behavior*.’ ([31] p. 182). In short, there is no temperature threshold to distinguish between normal and fragile personalities, and a psychodynamic approach in HH seems to be a fitting option [32].

## From ‘habitual hyperthermia’ to ‘functional hyperthermia’ and vice versa

HH has likely undergone a transformation similar to that of Beard’s neurasthenia [33, 34]. Nonetheless, here is a brief overview of the evolution of medical terminology.

In 1935, Moschcowitz introduced the term ‘*psychnosia*’ ([35] p. 603) to cover the field of functional disorders. Reimann’s cases are consistent with Moschcowitz’s hypotheses,^8^ although the claim that the symptoms take root after puberty is questionable. In fact, HH refers to two competing notions: hyperthermia has a physiological meaning, while the term ‘habitual’ has a psychological connotation [36]. This vocabulary thus mixes both experimental findings and a reinterpretation, if not an overinterpretation, of signs. This duality emerges in Wunderlich’s seminal text [37, 38]. In 1868, Wunderlich judged that the course of temperature was influenced by individual conditions on characterological grounds, as recalled in this passage:

In some individuals (healthy in other respects) of greater delicacy, especially women and children, the mobility of temperature is somewhat greater, and under corresponding conditions the vibrations may somewhat exceed the above limits (i.e. 100.4°F) ([37] p. 95).

Furthermore, despite his colossal work, Wunderlich devoted only a small chapter to neuroses and used the term ‘vaso-motor neuroses’ (*vasomotorische Neurosen*) ([37] p. 424) to designate transient, low-grade hyperthermia and continuous, more intense hyperthermia, or ‘hysterical fever,’ with no experimental evidence [39].^9^ However, the observations of Cawadias, Falcon-Lesses and Proger,^10^ Kintner and Rowntree, Smith, MacNeal, and Rappaport (to mention just a few), despite some bias, still offer important lessons from the past on psychogenic fever [40–45]. First, emotion is not a constant cause of a febrile response, and localised temperature elevations should not be overinterpreted. Then, a stressful situation can increase body temperature, but the reaction is nonlinear. Finally, a temperature measurement per se has no meaning without a correlation with the degree of complaint of the patient. Moreover, the meaning of ‘normal’ varies from normative, clinical, and statistical points of view, and a normal temperature for one individual may be abnormal for another [11, 46]. For this reason, it is important to repeat that the tipping point to a morbid state is the patient’s experience and level of distress [47]. On the other hand, in light of the evidence accumulated in the Japanese literature [48–50], Babinski’s repudiation of the ‘*reality of hysterical fever*’ ([51] p. 9) in the early part of the twentieth century must be balanced.

In 1909, Babinski claims that unexplained physical symptoms are fictitious if they are ‘*not likely to be induced or cured by suggestion*.’ ([51] p. 81). In answering Binet and Simon’s questions, Babinski even states that ‘*the possibility of creating vasomotor disorders by suggestion*’ ([52] p. 85) is impossible. The striking finding in the contemporary work of Hiramoto et al. is that the febrile sensation could be triggered by heterosuggestion, confirming the older assumptions of Eichelberg and von Eiff [48, 53, 54]. Besides, even though the adolescent’s oral temperature remained below the definition of ‘hyperthermia’ (i.e. < 99.9°F), unlike Eichelberg’s patient, the case also highlights Canguilhem’s warning:

The borderline between the normal and the pathological is imprecise for several individuals considered simultaneously but it is perfectly precise for one and the same individual considered successively (Fawcett CR, trans, 1943/1991) ([11] p. 184).

**Fig. 1:**
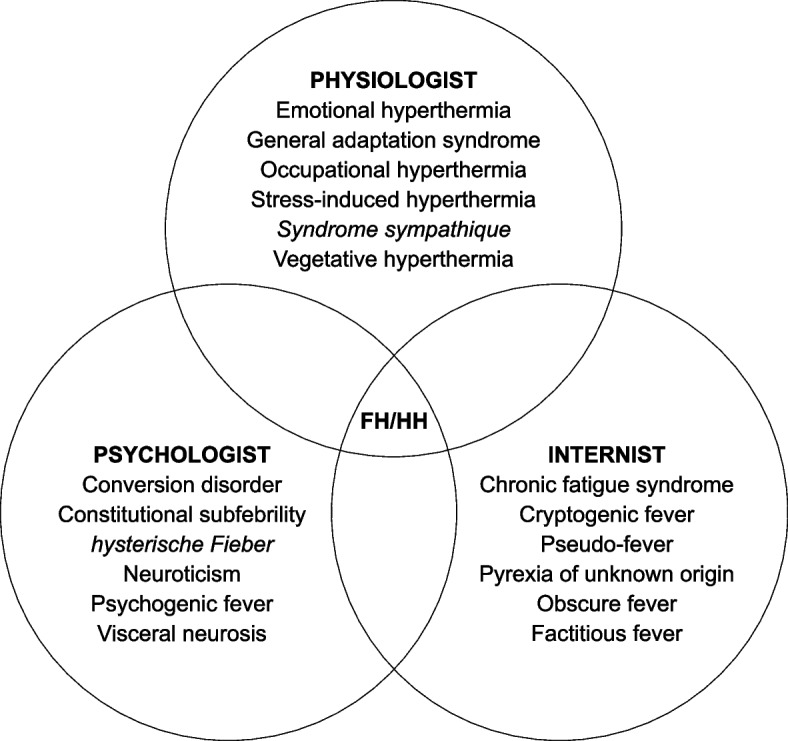
Habitual hyperthermia-like concepts. *Abbreviations*: FH, functional hyperthermia; HH, habitual hyperthermia. *Notes*: This list is not exhaustive

In 1987, Kimura et al. arbitrated that HH ‘*is the most representative cause of functional slight fever*’ ([55] p. 138) among ‘nonorganic diseases.’ More recently, in 2015, Oka suggested using the term ‘functional hyperthermia’ for ‘psychogenic fever’ to avoid dualistic thinking, to emphasise neural mechanisms, and to separate complex cases from emotional hyperthermia in healthy subjects [56]. In fact, the term ‘functional’ is polysemous and has conflicting definitions throughout the history of psychiatry [57]. Furthermore, Bell et al. assert that the functional-organic distinction is too static and somehow influences the prestige of the symptoms [58]. However, FH is a relevant concept, as it refers to a condition that is (1) multicausal, (2) erratic, (3) precipitated by psychosocial factors, (4) without a specific biological signature, and (5) accessible to nonpharmacological care. In addition, the concept helps to overcome the ‘substantialist obstacle,’ the belief that each diagnosis relies on a single biological anomaly [59, 60]. Figure 1 is an overview of terms close to HH [12, 17–19, 40–45, 49, 53, 55, 56].

## Diagnostic considerations

FH/HH is at the crossroads of three major disciplines: medicine, psychology, and physiology. It is tempting to assert that FH and HH are a part of a general functional syndrome and are not entities [61, 62]. However, this concern for unification does not eliminate the difficulty of determining which symptom is specific and which is not, nor the polymorphism of febrile illnesses [63]. In his major work on stress, Selye admitted ‘*that specificity is always a matter of degree. Both among changes and among causes, there are fluent transitions between the least and the most specific*.’ ([64] p. 56). Therefore, the diagnostic challenge in cases of fever without apparent focus and clinical distress should be to reduce uncertainty while limiting the risk of harmful interventions. Moreover, philosopher Peirce suggests^11^ the following:

[L]ogicians should have two principal aims: 1st, to bring out the amount and kind of security (approach to certainty) of each kind of reasoning, and 2nd, to bring out the possible and esperable uberty, or value in productiveness, of each kind ([65] p. 248).

In real-life clinical practice, Peirce's pragmatism calls for selecting a limited number of hypotheses. Obviously, a diagnostic error might occur, but it could be reduced with proper follow-up. Hence, the examination must be attentive to the patient’s anxiety level and establish whether (1) a measurement error or artefact is plausible, (2) a drug may be involved, or (3) a functional aetiology can be retained after minimal testing. Table 2 recalls the diagnostic criteria for HH proposed by Knockaert and Bobbaers in the 1990s [66]. The list of symptoms may be amended by the experience of general discomfort, dizziness, or even interference with the patient’s social life, but a ‘*belle indifférence*’ should not be misinterpreted [67–69].

**Table 2:** Belgian (University Hospitals Leuven) diagnostic criteria for ‘habitual hyperthermia’ in 1990

1. Age 16 to 40 years with no immunosuppression or drug dependence
2. Axillary temperature less than or equal to 101.3°F (38.5 °C)
3. Increased body temperature after mild exertion
4. No effect of antipyretics
5. Additional functional symptoms include fatigue
6. No localising signs
7. No laboratory abnormalities (i.e. CBC w/diff, ESR, APP, s-TSH, ANA, urinalysis)
8. Normal chest X-ray and abdominal ultrasound
9. No alternative hypotheses

Redrawn from Knockaert and Bobbaers, with the authorisation and courtesy of the journal [66]

*Abbreviations*: ANA, antinuclear antibodies; APP, acute-phase proteins; CBC, complete blood count; ESR, erythrocyte sedimentation rate; TSH, thyroid stimulating hormone

**Fig. 2:**
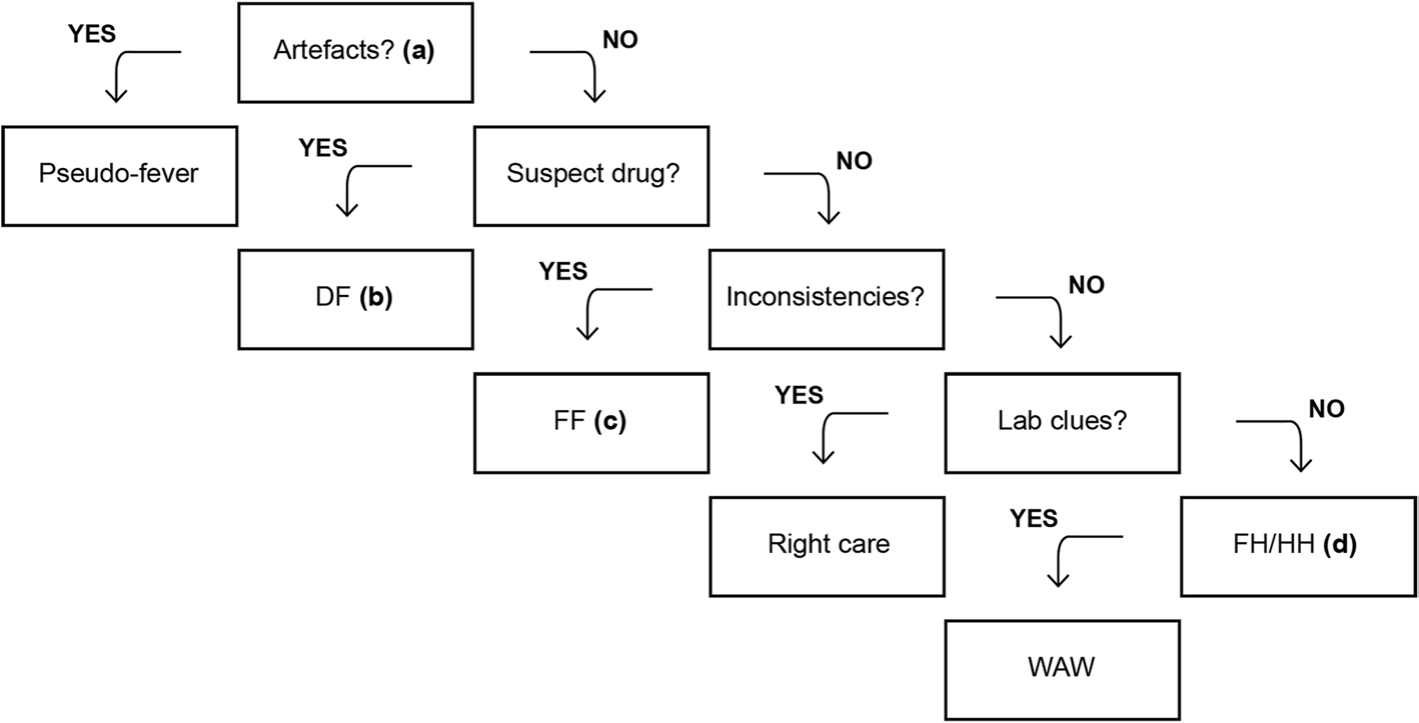
Algorithmic approach to an ‘inappropriately well’ adult with the complaint of persistent fever. *Abbreviations*: DF, drug-induced fever; FF, factitious fever; FH, functional hyperthermia; HH, habitual hyperthermia; WAW, watch-and-wait. *Notes*: **a** Thermometer placement, circadian variation, luteal phase, physical activity, chewing, smoking, caffeine, sleep patterns. **b** Fever is usually hectic, and biological signs are inconsistent. Only a resolution of symptoms within 72 h after discontinuation of treatment, makes the diagnosis probable [72]. **c** Other clues: health care personnel, peregrination, undocumented fever, hyperpyrexia, psychiatric comorbidities, and inadequate urinary temperature [73, 74]. **d** The entire debate concerns the value given to lab tests and particularly to inflammatory biomarkers, whose performance is poor in nonhospital-based medicine. Serum protein electrophoresis seems to be more useful for detecting an inflammatory pattern

Ultimately, even though chronic biological inflammation must be ruled out, it is advised to place more weight on the medical examination than on specific biomarkers [1, 55]. Cunha et al. also noted in a clinical approach to persistent fever that the ‘*diagnostic specificity of nonspecific laboratory abnormalities is increased when considered together*.’ ([70] p. 5). Naturally, if the patient looks ‘inappropriately well,’ it might be challenging to confirm that the fever is genuine. A solution might be a fever tracker app or calendar, but it is crucial to take into account the possibility that self-measurement may make symptoms worse. Therefore, the follow-up in cases of suspicion of FH/HH should be brief. Affronti et al. suggest a reassessment every two months and biological control at six months [68]. In their experience, only 3% of patients had a misdiagnosis of HH beyond this period. Figure 2 provides an algorithm^12^ to identify FH/HH among drug fevers and factitious disorders, which Vanderschueren and Knockaert categorise as ‘*little three entities*’ ([71] p. 412) in the aetiological spectrum of pyrexia of unknown origin.

## Conclusion

‘Habitual hyperthermia’ is not an obsolete entity and forces the clinician to explore nonstandard possibilities. However, differential diagnoses, such as circadian temperature rhythm, iatrogenesis, malingering, and above all, incomplete history-taking, must not be neglected. Thus, in the absence of clear signs, tests should be performed with tact, and measurement and medical reassessment should be the primary focus to avoid misdiagnosis. Finally, yet importantly, Canguilhem’s theories on normality should continue to be a guide for patient-centred care.

## Data Availability

Not applicable.

## Footnotes

1 The issue of diagnostic work-ups in cases of persistent fever without localising signs was debated in a previous article. However, the philosophical problems about ‘normality’ have been ignored, as has the historical evolution of the ‘habitual hyperthermia’ concept. This second paper focuses on nosographic difficulties.

2 Hobart Ansteth Reimann (1897–1986) was an American physician best known for his description of periodic disease in 1947.

3 ‘*The criteria selected* [for a case of prolonged unexplained fever] *were*: … *Fever higher than 101°F on several occasions. This eliminated the entity of habitual hyperthermia*.’ ([9] p. 2).

4 Given the risk of diverting from the topic, the argument that the issue with medically unexplained symptoms (MUS) is fundamentally economic rather than terminological could not be developed.

5 Moro appears to be referring to Ernst Kretschmer (1888–1964), although this psychiatrist is not mentioned.

6 The text does not address the legitimacy of the concept of ‘neurosis,’ which has both a descriptive and an etiological meaning and which was partially abandoned by the American Psychiatric Association in the 1980s, but rather the taxonomic difficulties revealed by Reimann’s series.

7 Historical figure of the American feminist movement, patient of Dr. Silas Weir Mitchell (1829–1914).

8 According to Moschcowitz, functional disorders are strictly human ‘diseases,’ developing after puberty, punctuated by social crises (like the Great Depression) and recurring throughout life. His formula of ‘*exaggerations of normal function*’ ([35] p. 611) echoes Rosenbach's (1851–1907) nineteenth century work and the continuity between a functional disorder and a structural lesion.

9 As stated by Faber, Wunderlich finally accepted ‘*that the various diseases ran typical courses*’ ([39] p. 22) and opted, in spite of his criticism of the French naturalists, for a constructivist ontology.

10 Even though Falcon-Lesses and Proger noted in Miss A. V. an increase in body temperature of 1.4°F (0.8 °C) following a venipuncture, considering her 44 hospital visits, it is hard to claim that she was of a ‘*calm, equable disposition and never objectively manifested any signs of emotional disturbance*.’ ([41] p. 1035).

11 Letter to Frederick Adam Woods, 1913.

12 Obviously, diagnoses of drug-induced fever, factitious fever, or FH/HH can only be made after several consultations, and an algorithm cannot address the complexity of care. Thus, the figure is more illustrative. The question of whether to increase the number of tests, that is, whether or not to tolerate diagnostic uncertainty, was not addressed in the article. On the other hand, it seems evident that the subject of MUS extends beyond simple taxonomic considerations. Any diagnosis is contingent upon (1) medical experience, (2) the actual availability of tests, and (3) the pressures to induce demand. However, what differentiates hospital medicine from family medicine is the level of therapeutic empiricism. In family medicine, most diagnoses are made *ex juvantibus*.

## References

[1] Ginier-Gillet M, Esparcieux A (2021). Habitual hyperthermia: an interpretive paradigm of the 20th century? Not really. Int J Gen Med.

[2] Knockaert DC, Dujardin KS, Bobbaers HJ (1996). Long-term follow-up of patients with undiagnosed fever of unknown origin. Arch Intern Med.

[3] Vickery DM, Quinnell RK (1977). Fever of unknown origin: an algorithmic approach. JAMA.

[4] Yang OO, Currier JS (2016). Reimann’s “habitual hyperthermia” responding to hormone therapy. Open Forum Infect Dis.

[5] Konecny P, Davidson RN (1996). Pyrexia of unknown origin in the 1990s: time to redefine. Br J Hosp Med.

[6] Wright WF, Simner PJ, Carroll KC, Auwaerter PG (2022). Progress report: next-generation sequencing (NGS), multiplex polymerase chain reaction (PCR), and broad-range molecular assays as diagnostic tools for fever of unknown origin (FUO) investigations in adults. Clin Infect Dis.

[7] Katon WJ, Walker EA (1998). Medically unexplained symptoms in primary care. J Clin Psychiatry.

[8] Reimann HA (1947). Regularly periodic fever of eleven years‘ duration: report of a case. Acta Med Scand.

[9] Petersdorf RG, Beeson PB (1961). Fever of unexplained origin: report on 100 cases. Medicine (Baltimore).

[10] Spicker SF (1987). An introduction to the medical epistemology of Georges Canguilhem: moving beyond Michel Foucault. J Med Philos.

[11] Debru C, Dupont JC, Fagot-Largeault A, Lambert J, Schmidgen H, editors. Georges Canguilhem. OEuvres complètes tome II. Écrits de médecine et de philosophie : Les thèses [Collected papers of Georges Canguilhem volume II. Writings in medicine and philosophy: The doctoral dissertations]. Paris: Librairie philosophique J. Vrin; 2021. French.

[12] Moro E. Habituelle Hyperthermie [Habitual hyperthermia]. In: Keller-Berlin A, editor. Monatsschrift für Kinderheilkunde, Band XIV. Leipzig, Wien: Franz Deuticke; 1918. p. 214–23. German.

[13] Neff FC (1922). Temperature variability in certain apparently healthy children. South Med J.

[14] Finkelstein H (1924). Habitual hyperthermia during recovery from scarlet fever. J Am Med Assoc.

[15] Brünecke K (1926). Über habituelle Hyperthermie [About habitual hyperthermia]. Beiträge zur Klinik der Tuberkulose..

[16] Mackowiak PA, Wasserman SS, Levine MM (1992). A critical appraisal of 98.6°F, the upper limit of the normal body temperature, and other legacies of Carl Reinhold August Wunderlich. JAMA.

[17] Reimann HA (1932). Habitual hyperthermia. J Am Med Assoc.

[18] Reimann HA (1935). Habitual hyperthermia: a clinical study of four cases with long continued low grade fever. Arch Intern Med (Chic).

[19] Reimann HA (1936). The problem of long continued, low grade fever. J Am Med Assoc.

[20] Holló J, Holló-Weil E. Experimentelle Analyse der subfebrilen Temperaturen und ihre Ergebnisse [Experimental analasyis of low grade fever and their results]. Berl Klin Wochenschr. 1918;55:640–3. German.

[21] Menninger K (1944). The abuse of rest in psychiatry. J Am Med Assoc.

[22] Gildea MCL, Gildea EF. Personalities of American psychotherapists: Mitchell, Salmon, Riggs. Am J Psychiatry. 1945;101:460–7. 10.1176/ajp.101.4.460.

[23] White KL, Long WN (1958). The incidence of psychogenic fever in a university hospital. J Chronic Dis.

[24] Mold JW, Stein HF (1986). The cascade effect in the clinical care of patients. N Engl J Med.

[25] Micale MS (1989). Hysteria and its historiography: a review of past and present writings (II). Hist Sci.

[26] Freud S. L’hérédité et l’étiologie des névroses [Heredity and the aetiology of the neuroses]. Rev Neurol (Paris). 1896;4:161–9. French.

[27] Knoff WF (1970). A History of the concept of neurosis, with a memoir of William Cullen. Am J Psychiatry.

[28] Janet P. Les névroses [Neuroses]. Paris: Flammarion; 1909. French.

[29] Gilman CP. Why i wrote the yellow wallpaper? Forerunner. 1913;IV:271.

[30] Knight DD. “All the facts of the case”: Gilman’s lost letter to Dr. S. Weir Mitchell. Am Lit Realism. 2005;37:259–77.

[31] Kubie LS (1954). The fundamental nature of the distinction between normality and neurosis. Psychoanal Q.

[32] Meyer R, Beck D (1976). Zur Psychodynamik des psychogenen Fiebers [Psychodynamics of psychogenic fever]. Z Psychosom Med Psychoanal.

[33] Straus SE (1991). History of chronic fatigue syndrome. Rev Infect Dis.

[34] Micale MS. On the “disappearance” of hysteria: a study in the clinical deconstruction of a diagnosis. Isis. 1993;84:496–526. 10.1086/356549. 10.1086/356549 8282518

[35] Moschcowitz E (1935). The psychogenic origin of organic diseases. N Engl J Med.

[36] Prince M (1898). Habit neuroses as true functional diseases. Boston Med Surg J.

[37] Wunderlich KRA. On the temperature in diseases: A manual of medical thermometry. Woodman Bathurst W, translator. 2nd Ed. London: New Sydenham Society; 1871.

[38] Mackowiak PA, Worden G (1994). Carl Reinhold August Wunderlich and the evolution of clinical thermometry. Clin Infect Dis.

[39] Faber K (1922). Nosography in modern internal medicine. Ann Med Hist.

[40] Cawadias A (1920). La fièvre continue d’origine sympathique [Continuous fever of sympathetic origin]. Ann De Med.

[41] Falcon-Lesses M, Proger SH (1930). Psychogenic fever. N Engl J Med.

[42] Kintner AR, Rowntree LG (1934). Long continued, low grade, idiopathic fever: analysis of one hundred cases. J Am Med Assoc.

[43] Smith DS (1939). Fever of undetermined etiology. J Mich State Med Soc.

[44] MacNeal WJ (1939). Hyperthermia, genuine and spurious. Arch Intern Med (Chic).

[45] Rappaport EM. Essential oral hyperthermia; report of a study of 25 cases of low grade ‘fever.’ Ann Intern Med. 1946;25:1–14. 10.7326/0003-4819-25-1-1. 10.7326/0003-4819-25-1-1 20991213

[46] Winckelmann G, Maass G, Schmidt H, Löhner J (1986). Vegetative Hyperthermie: Thermoregulationsstörung oder Variante der Norm? [Vegetative hyperthermia: a thermoregulation disorder or a variant from the norm?]. Dtsch Med Wochenschr..

[47] Minkowski E. Le temps vécu : Études phénoménologiques et psychopathologiques [Lived time: Phenomenological and psychopathological studies]. Reprint. Paris: PUF; 1933/2013. French.

[48] Hiramoto T, Oka T, Yoshihara K, Kubo C (2009). Pyrogenic cytokines did not mediate a stress interview-induced hyperthermic response in a patient with psychogenic fever: a case report. Psychosom Med.

[49] Oka T. Stress-induced hyperthermia and hypothermia. In: Romanovsky AA, editor. Handbook of clinical neurology. 2018;157:599–621. 10.1016/B978-0-444-64074-1.00035-5 10.1016/B978-0-444-64074-1.00035-5 30459027

[50] Nakamura K, Morrison SF (2022). Central sympathetic network for thermoregulatory responses to psychological stress. Auton Neurosci.

[51] Babinski J. Démembrement de l’hystérie traditionnelle : Pithiatisme [The dismemberment of traditional hysteria: Pithiatism]. Paris: J Charpentier; 1909. French.

[52] Binet A, Simon T (1909). Hystérie [Hysteria]. Annee Psychol.

[53] Eichelberg F (1921). Durch Hypnose erzeugtes “Hysterisches Fieber” [“Hysterical fever” induced by hypnosis]. Dtsch Z Nervenheilkd..

[54] von Eiff AW (1951). Der Einfluß der Hypnose auf Temperaturempfindung und Wärmeregulation [The influence of hypnosis on thermosensation and thermoregulation]. Z Gesamte Exp Med..

[55] Kimura M, Tano Y, Tomizawa S, Hirano Y (1987). Clinical study on slight fever. Kawasaki Med J..

[56] Oka T (2015). Psychogenic fever: how psychological stress affects body temperature in the clinical population. Temperature (Austin).

[57] Beer MD (1996). The dichotomies: psychosis/neurosis and functional/organic: a historical perspective. Hist Psychiatry.

[58] Bell V, Wilkinson S, Greco M, Hendrie C, Mills B, Deeley Q (2020). What is the functional/organic distinction actually doing in psychiatry and neurology?. Wellcome Open Res..

[59] Bachelard G. La formation de l’esprit scientifique : Contribution à une psychanalyse de la connaissance objective [The formation of the scientific mind: A contribution to a psychoanalysis of objective knowledge]. 5th Ed. Paris: Librairie philosophique J. Vrin; 1967. p. 97–130. French.

[60] Fuller J (2018). Universal etiology, multifactorial diseases and the constitutive model of disease classification. Stud Hist Philos Biol Biomed Sci.

[61] Hasan MK, White AC (1979). Psychogenic fever: entity or nonentity?. Postgrad Med.

[62] Wessely S, White PD (2004). There is only one functional somatic syndrome. Br J Psychiatry.

[63] Horowitz HW (2013). Fever of unknown origin or fever of too many origins?. N Engl J Med.

[64] Selye H (1956). The stress of life.

[65] Burks AW, editor. Collected papers of Charles Sanders Peirce. Volume VIII: Reviews, correspondence, and bibliography. Cambridge: Harvard University Press; 1958. p. 246–8.

[66] Knockaert DC, Bobbaers H (1990). Geneesmiddelenkoorts en habituele hyperthermie [Drug fever and habitual hyperthermia]. Tijdschr Geneeskd.

[67] Stone J, Smyth R, Carson A, Warlow C, Sharpe M (2006). *La belle indifférence* in conversion symptoms and hysteria: systematic review. Br J Psychiatry.

[68] Affronti M, Mansueto P, Soresi M (2010). Low-grade fever: how to distinguish organic from non-organic forms. Int J Clin Pract.

[69] Kunimatsu J (2020). Clinical aspects of functional hyperthermia. Jpn J Psychos Med.

[70] Cunha BA, Lortholary O, Cunha CB (2015). Fever of unknown origin: a clinical approach. Am J Med.

[71] Vanderschueren S, Knockaert DC. Tackling fever and inflammation of unknown origin: the do’s and don’ts. Acta Clin Belg. 2014;69:412–7. 10.1179/2295333714Y.0000000070. 10.1179/2295333714Y.0000000070 25176406

[72] Patel RA, Gallagher JC (2010). Drug fever. Pharmacotherapy.

[73] Wallach J (1994). Laboratory diagnosis of factitious disorders. Arch Intern Med.

[74] Galli S, Tatu L, Bogousslavsky J, Aybek S. Conversion, factitious disorder and malingering: a distinct pattern or a continuum? In: Bogousslavsky J, editor. Neurologic-psychiatric syndromes in focus. Part II: From psychiatry to neurology. Front Neurol Neurosci. 2018;42:72–80. 10.1159/000475699 10.1159/000475699 29151092

